# Food web assessments in the Baltic Sea: Models bridging the gap between indicators and policy needs

**DOI:** 10.1007/s13280-021-01692-x

**Published:** 2022-01-29

**Authors:** Samuli Korpinen, Laura Uusitalo, Marie C. Nordström, Jan Dierking, Maciej T. Tomczak, Jannica Haldin, Silvia Opitz, Erik Bonsdorff, Stefan Neuenfeldt

**Affiliations:** 1 Finnish Environment Institute, Latokartanonkaari 11, 00790 Helsinki, Finland; 2 Åbo Akademi University, BioCity, 20500 Turku, Finland; 3 GEOMAR, Helmholtz Centre for Ocean Research Kiel, Duesternbrooker Weg 20, 24105 Kiel, Germany; 4 Baltic Sea Centre, Stockholm University, 106 91 Stockholm, Sweden; 5 HELCOM Secretariat, Katajanokanlaituri 6B, 00160 Helsinki, Finland; 6 National Institute of Aquatic Resources, Technical University of Denmark (DTU Aqua), Kemitorvet, 2800 Kgs. Lyngby, Denmark

**Keywords:** Baltic Sea, Ecosystem-based management, Food web assessment, Food web indicators, Food web models, Marine Strategy Framework Directive

## Abstract

Ecosystem-based management requires understanding of food webs. Consequently, assessment of food web status is mandatory according to the European Union’s Marine Strategy Framework Directive (MSFD) for EU Member States. However, how to best monitor and assess food webs in practise has proven a challenging question. Here, we review and assess the current status of food web indicators and food web models, and discuss whether the models can help addressing current shortcomings of indicator-based food web assessments, using the Baltic Sea as an example region. We show that although the MSFD food web assessment was designed to use food web indicators alone, they are currently poorly fit for the purpose, because they lack interconnectivity of trophic guilds. We then argue that the multiple food web models published for this region have a high potential to provide additional coherence to the definition of good environmental status, the evaluation of uncertainties, and estimates for unsampled indicator values, but we also identify current limitations that stand in the way of more formal implementation of this approach. We close with a discussion of which current models have the best capacity for this purpose in the Baltic Sea, and of the way forward towards the combination of measurable indicators and modelling approaches in food web assessments.

**Supplementary Information:**

The online version contains supplementary material available at 10.1007/s13280-021-01692-x.

## Introduction

With increasing human use of the marine environment, an ecosystem-based approach to management of human activities is widely acknowledged as the fundamental principle to accomplish sustainable resource use and maintain healthy marine ecosystems (Pikitch et al. 2004; McLeod and Leslie 2009). The underlying aim of this approach is an ecologically sound resource management that responds to natural ecosystem processes (Marasco et al. 2007). Ecological indicators, serving as proxies for multiple ecological processes and representing ecosystem states, are being proposed to better inform management decisions. Of particular interest are food web indicators, which are becoming increasingly important because they inform of the state of marine ecosystem functionality for various policy needs (e.g. Rombouts et al. 2013; Broszeit et al. 2017) and closely link with central ecosystem services (Longo et al. 2015).

The critical and presently unresolved step in the science-policy process is to identify and agree on food web indicators that are not only understandable and defensible to all stakeholders, but also capture key food web states and processes that underlie critical and complex ecosystem dynamics (Tam et al. 2017). This complexity is difficult to monitor, as food webs are temporally and spatially dynamic, and comprised of highly diverse and interconnected components (Kortsch et al. 2021). The EU Marine Strategy Framework Directive (MSFD; EU 2008) and the revised EU Common Fisheries Policy (CFP) recognize this and call for better integration of food web characteristics in the assessment and management of biological resources.

Whereas the European Union’s nature directives address structure and functions of habitats or populations (e.g., EU 1992), only the MSFD includes special attention to food webs, being one of the eleven descriptors for ‘good environmental status’ (GES). In 2018, all EU Member States provided reports on the state of the marine environment, which were found to include surprisingly few food web indicators (https://water.europa.eu/marine). With food web indicators, we mean any data product that can be used to assess the state of a marine food web and that reflects impacts of human activities. In this perspective, we provide a comprehensive review and a gap analysis of the food web indicators developed for the Baltic Sea, which serves as a good example of a marine area with a well-established and long-running monitoring regime and is one of the most data-dense regions in the world. Many indicators have been operationalized, and knowledge of the food web interactions is on a comparatively good basis due to the relatively low marine diversity compared to oceanic ecosystems (HELCOM 2018). Despite this good starting point, we show that the indicator suite is strongly focused on the state of populations and has not been developed to support assessments of interconnected components (i.e. not considering food web interactions). Moreover, there is very little evidence that the GES is defined in coherence among the food web indicators (however, see Kauhala et al. 2019). Altogether, this means that despite the recent interest to advance and conduct food web assessments in the Baltic Sea, a feasible, widely applicable, holistic and ecologically relevant approach to design and carry out food web assessments in the region is still lacking. To support the development and ultimately implementation of such an approach, we then reviewed a portfolio of scientifically published ecosystem models for the same area and assessed their usefulness to help integrating the complexity of food webs in the assessments. Finally, we evaluated the capacity of the models to aid indicator-based assessment of food web status. As the Baltic presents an ideal test case, we argue that the lessons learned are generally applicable.

## Food web indicators in the Baltic Sea: State-of-the-art

To summarize the state-of-the-art regarding all food web indicators for the Baltic Sea, we compiled them from three sources of indicator information: the EU member states’ reporting under the MSFD in 2018 (https://water.europa.eu/marine), the HELCOM indicator catalogue (https://helcom.fi/baltic-sea-trends/indicators/), as well as scientific publications of food web indicators. The scientific publications were available through multiple, properly documented international sources, including research papers in scientific journals, EU-funded project reports, and online publications by international organisations or research groups (for further details see Ojaveer et al. 2020).

The search was concluded with a total of 64 hits and, after removing four clear overlaps, we identified 60 food web-related indicators with different application areas in the Baltic Sea (see full results in Appendix A). Some of the indicators were closely similar, but we treated them separately if there were differences in the name or calculation method and the descriptions did not mention similarity to any other indicator.

In this study, we use the requirements of the MSFD, because this offers a well-defined, legal basis for the analysis. Although the MSFD requirements pertain only to the EU member states, definitions of the food web assessments are applicable to all marine food webs. The MSFD defines GES of food webs by a qualitative descriptor 4 (D4), which is further divided into four GES criteria: the diversity within and balance of abundance between trophic guilds (i.e., the two primary criteria D4C1 and D4C2), as well as the two secondary GES criteria: the size distribution of individuals across a trophic guild (D4C3) and the productivity of the trophic guild (D4C4; Table 1). We categorized the indicators to these criteria from the reported information (Table 2). In addition, we noted the Baltic Sea sub-basins where each indicator is in use or was tested for, and identified which trophic guilds the indicators address using the list of trophic guilds by ICES (2015) and assigning species to trophic guilds following Ojaveer et al. (2020) (see Appendix A—Table S1). This synthesis allowed us to then conduct a gap analysis of the suite of available indicators against the GES criteria (Table 1).

**Table 1:** Information on the EU MSFD criteria for good environmental status of descriptor 4 ‘food webs’ (EU 2017). Specifications for an assessment are given for each criterion on the basis of the Commission Decision (EU 2017) (denoted as *) or based on our own criterion to calculate any distribution (†)

Criteria for marine food web assessments	
D4C1—Primary: The diversity (species composition and their relative abundance) of the trophic guild is not adversely affected due to anthropogenic pressures	
(†) There are 3 or more components (typically species) included in a trophic guild	
D4C2—Primary: The balance of total abundance between the trophic guilds is not adversely affected due to anthropogenic pressures	
(*) There are 3 or more trophic guilds in the model, including two non-fish and one primary producer guild	
D4C3—Secondary: The size distribution of individuals across the trophic guild is not adversely affected due to anthropogenic pressures	
(†) There are 3 or more age / size groups included in a trophic guild, or explicit modelling of mean weight, weight-at-age or similar	
D4C4—Secondary (to be used in support of criterion D4C2, where necessary): Productivity of the trophic guild is not adversely affected due to anthropogenic pressures	
(†) Parameters for reproduction rate, or the adult population and offspring production rate can vary in the model (to evaluate changes in productivity of the species)

**Table 2:** Availability of food web indicators by trophic guilds (ICES 2015) and the EU Marine Strategy Framework Directive (MSFD) criteria for good environmental status (GES). Geographical coverage of the indicators depicted from the indicator sources where the indicator is either operationally used or successfully tested. EU Member states reported the use of food web indicators in 2018 (https://water.europa.eu/marine). Criteria codes as in Table 1. Full indicator list in Appendix A

	Number of indicators	Addresses GES criteria				Sub-basins	Reported under MSFD
		D4C1	D4C2	D4C3	D4C4		
Primary producers: phytoplankton	7	4	4	0	0	All	DK, FI, LT, PL
Primary producers: macrophytes	0	0	0	0	0		
Secondary producers: zooplankton	9	4	5	1	0	All	DK, FI, LT, PL
Filter-feeders: benthos	0	0	0	0	0		
Deposit-feeders: benthos	2	2	0	0	0	All	PL
Planktivores: benthos	0	0	0	0	0		
Planktivores: nekton (excl. warm-blooded)	12	2	6	4	0	All	EE, LT, PL, SE
Planktivores: seabirds	3	2	3	0	0	All	DK
Planktivores: marine mammals	0	0	0	0	0		
Sub-apex pelagic predators: nekton (excl. warm-blooded)	19	3	9	5	2	All	DK, EE, LT, PL, SE
Sub-apex pelagic predators: seabirds	4	2	4	0	0	All	DK
Sub-apex pelagic predators: marine mammals	0	0	0	0	0		
Sub-apex demersal predators: benthos	0	0	0	0	0		
Sub-apex demersal predators: nekton (excl. warm-blooded)	16	3	7	5	0	All	DK, EE, LT, PL, SE
Sub-apex demersal predators: seabirds	4	2	4	0	0	All	DK
Sub-apex demersal predators: marine mammals	0	0	0	0	0		
Apex predators: nekton (excl. warm-blooded)	0	0	0	0	0		
Apex predators: seabirds	2	0	1	0	1	All	FI, PL
Apex predators: marine mammals	12	0	5	1	7	All	DK, FI, PL
Unspecified	1	0	1	0	0	EE marine area	EE

Country codes: DK = Denmark, EE = Estonia, FI = Finland, LT = Lithuania, PL = Poland, SE = Sweden

Table 2 shows that the majority of food web indicators fell into the two primary GES criteria (13 and 25, respectively), but that indicators were also found for the two secondary criteria (7 and 10 indicators, respectively). Some indicators covered two GES criteria. From the trophic guild point of view, 11 out of 19 trophic guilds were covered by the indicators. Significant gaps were found for macrophytes, benthic filter-feeders, benthic planktivores and sub-apex benthic invertebrate predators for which no indicators were found (Table 2). Planktivorous mammals, demersal mammals and apex fish predators also lacked indicators, but these guilds do not exist in the region. There were 15 indicators for planktonic guilds, but a closer look shows that these do not include jellies or mysids which are important elements of food webs in large parts of the Baltic (Hays et al. 2018; Kiljunen et al. 2020). Benthic deposit feeders were covered by two indicators. Altogether 12 indicators were found for planktivorous fish, and 16 and 19 indicators for sub-apex predatory fish of demersal and pelagic species, respectively. Two seabird indicators addressed apex predators, three indicators for planktivores and four indicators for pelagic and demersal sub-apex predator each. Marine mammals were covered by 12 indicators.

The balance between trophic guilds (i.e. D4C2) is an essential indication of the state of food webs. It means that the biomass or abundance ratios of the different trophic guilds are not adversely affected, as might happen for example if the abundance of top predators decreases and that of their prey, therefore, increases. We found that 11 indicators include at least three guilds (as required by the MSFD, see Table 2). However, the majority do not assess the balance between the guilds per se but rather give separate results for each guild; i.e. GES is not assessed for the balance of the guilds. The only indicators truly bridging trophic levels (i.e., directly providing for D4C2) are the following: *Ratio of total zooplankton biomass to total phytoplankton biomass, Balance of lower guilds, Zooplankton mean size and total stock (MSTS),* as well as the various indices comparing proportion of large fish to all the fish (e.g. *Large fish index* or *Fish community trophic index*, see Appendix A)*.* All these cover two trophic levels, except the MSTS which attempts to cover three levels, determining status on the basis of phytoplankton abundance (as food for zooplankton) and on the basis of the condition of planktivorous fish (indicating zooplankton as food for fish) (Gorokhova et al. 2016).

Based on the analysis, we claim that most of the indicators do not explicitly address the GES criteria. In the case of D4C1, D4C3 or D4C4, they do not indicate diversity, size distribution, or productivity within a trophic guild but within a taxonomic group. Similarly, under D4C2, they do not indicate balance between guilds, but abundance within a guild or a larger species group. These failures to meet the requirements are obviously caused by the ‘recycling’ of indicators from other state assessments to address food webs. It is, however, also clear that the indicators can rather easily be developed to operational indicators meeting the food web perspective by re-arranging the data, whereas re-defining indicator thresholds to set GES may require more elaboration.

The identified food web indicators have sufficient and relevant spatial coverage in the Baltic Sea (Table 2), but our analysis suggests that they only partly reflect changes that are caused by manageable pressures and their GES targets are not well defined (Table 3). The key requirements for such food web indicators include sensitivity to distinguish impacts of anthropogenic (manageable) pressures and certainty in defining GES (EU 2017). It seems obvious that there are severe gaps in the availability and application of the food web indicators in the Baltic Sea.

**Table 3:**
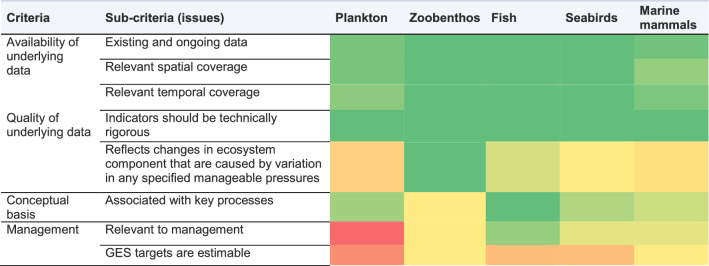
Rank-based evaluation of food web indicators for meeting the selected data and management-related criteria (Tam et al. 2017, adjusted). Green shades: generally meets criteria (darker shade means stronger agreement); yellow shades—meets criteria only partly, red—fails to meet criteria. The evaluation of GES thresholds was strictly evaluated against the needs of the MSFD criteria (EU 2017). See Appendix A for full evaluation

## Trophic models for the Baltic Sea and their application

One of the central objectives of this study was to evaluate whether food web models could fill the gaps in the current suite of food web indicators. We made a Web of Science search of the models using the keywords ‘model’, ‘food web’ and ‘Baltic Sea’ (incl. their various expressions). As the search terms were, on purpose, general, we received > 250 hits among which we had to pick studies fitting to at least one of the GES criteria (see Table 1). In addition, we excluded studies where Baltic food web models were used as components of a geographically or thematically larger study (e.g. Piroddi et al. 2021) or where different models were compared (e.g. Gårdmark et al. 2013). Finally, we identified 36 papers presenting suitable food web related models and included them in the present review (Table 4, Appendix 1). Some of the papers self-report that they build on models in earlier studies, but for consistency, we treated them all as separate models in this paper.

**Table 4:**
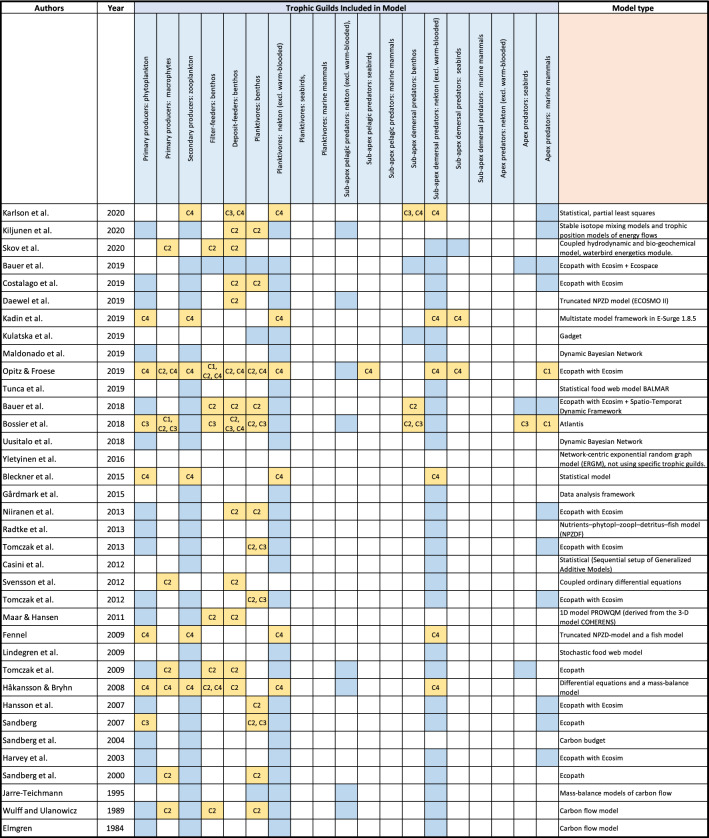
The trophic guilds each model includes (all coloured cells) and the model type. If the model has potential of filling an indicator gap for a trophic guild, that cell is coloured yellow and the criteria are shown. Note that only the potential for filling indicator gaps (identified in Table 2) are highlighted. Full details of the classification to GES criteria are given in Appendix A

Karlson et al. (2020), Kiljunen et al. (2020), Skov et al. (2020), Bauer et al. (2018, 2019), Costalago et al. (2019), Daewel et al. (2019), Kadin et al. (2019), Kulatska et al. (2019), Maldonado et al. (2019), Opitz and Froese (2019), Tunca et al. (2019), Bossier et al. (2018), Uusitalo et al. (2018), Yletyinen et al. (2016), Blenckner et al. (2015), Gårdmark et al. (2015), Niiranen et al. (2013), Radtke et al. (2013), Tomczak et al. (2009), Casini et al. (2012), Svensson et al. (2012), Maar and Hansen (2011), Fennel (2009), Lindegren et al. (2009), Håkansson and Bryhn (2008), Hansson et al. (2007), Sandberg (2007), Sandberg et al. (2000, 2004), Harvey et al. (2003), Jarre-Teichmann (1995), Wulff and Ulanowicz (1989), Elmgren (1984)

To evaluate whether the models can provide information that could be used to aid food web assessments, we categorized the models according to the GES criteria (see Table 1). Further, the Commission Decision (EU 2017) specifies that the selected trophic guilds meet these criteria: at least three trophic guilds (defined according to ICES 2015) are included, preferably from top, middle and bottom of the food chain, two of them are non-fish trophic guilds and at least one is a primary producer trophic guild. In this study, we call these ‘evaluation criteria’. Moreover, we defined two additional evaluation criteria for the assessments of diversity (D4C1) and size distribution (D4C3) that a minimum of three components are needed to assess those, and that an assessment of productivity (D4C4) requires a model to enable a varying reproduction rate (Table 1). We note that these two latter criteria are artificial, but they help to filter out models not suitable for these two GES criteria. We evaluated whether the models support the assessments with these conditions.

The models are mostly defined at species (sometimes genus or higher taxa) level. We mapped the species in each model to the trophic guilds according to ICES (2015) and Ojaveer et al. (2020) (Appendix A—Table S1). Most of the models included the three most important commercially exploited fish species, sprat, herring, and cod which represent the ‘Planktivores: nekton (excl. warm-blooded)’ and ‘Sub-apex demersal predators: nekton (excl. warm-blooded)’ (Table 4). Other trophic guilds that were often represented in the models included phytoplankton, zooplankton, and benthic planktivores (practically mysids). Marine mammal apex predators, most often seals, and in the Atlantis model also harbour porpoise, were represented in 13 models. The recent Ecopath with Ecosim (EwE, Bauer et al. 2018; Opitz & Froese 2019) and Atlantis (Bossier et al. 2018) models included the highest number of trophic guilds, 13, 10 and 12, respectively. Some of the models included modelled groups that could not be mapped into trophic guilds, such as meiozoobenthos, macrozoobenthos or ‘omnivores’. In the case of Yletyinen et al. (2015) the trophic guilds were not identified for this reason but in Sandberg et al. (2004), the group “fish” was assumed to include planktivorous fish, as this model was otherwise dealing with lower trophic levels only. In addition, our review includes information on the geographical areas that the models cover, the model type, and whether they can, based on the trophic guilds and taxa that they include, help to fill the gaps in the Baltic Sea food web indicator suite (Table 4, Appendix A—Table S4).

The analysis (Table 4) indicates that the models show potential for helping food web assessments particularly in relation to D4C2 (balance between trophic guilds) for benthos, where indicators were lacking. Some models could also help in assessing the D4C4 (productivity), while gaps in D4C1 (diversity of a trophic guild) were only filled by the models of Bossier et al. (2018) and Opitz and Froese (2019). The gaps in D4C3 (size distribution within trophic guild) were potentially filled by five models.

## How can models help in food web status assessments?

The big question is whether and how models have the potential to help with food web assessments in practice, and what currently stands in the way. For example, how do we move from an observed gap in a measurable indicator, to filling this gap based on model output? While we found gaps in the set of food web indicators in the Baltic Sea especially for macrophytes, birds and filter- and deposit-feeding benthos (Tables 2 and 3), these groups are included in eight, seven, and 14 of the 36 models, essentially the EwE model family (Tomczak et al. 2009, 2012, 2013; Bauer et al. 2018, 2019; Opitz & Froese 2019) as well as the Atlantis model (Bossier et al. 2018) (Table 4, Appendix A—Table S4). While all the existing models already include some of the key pressures on the Baltic Sea, most often fisheries and eutrophication, impacts of hazardous substances and non-indigenous species were missing in many models. The EwE and Atlantis models can be developed to include these pressures and evaluate their management (Fulton et al. 2011; Pinnegar et al. 2014; Piroddi et al. 2015; Walters and Christensen 2018).

The reliability and potential usefulness of model-derived time series (e.g. biomasses, catches or mortalities) to fill in the indicator gaps can be evaluated through reviewing model predictions for ecosystem components whose data has not been included into the model, but for which some data are available so that the prediction accuracy can be evaluated (e.g. Natugonza et al. 2020; however, see Maldonado et al. 2019 for caution). For example, Trifonova et al. (2015) created a dynamic Bayesian network model for the North Sea that was able to mimic zooplankton dynamics even though zooplankton data was not included into the model. Also EwE models have been shown to predict observed data relatively well at different trophic levels (Tomczak et al. 2012; Piroddi et al. 2017; Chagaris et al. 2020).

Also Tommasi et al. (2021) recommended that EwE and the Atlantis models can adequately capture the entire food web and support management decisions. The EwE models have great potential in providing multiple spatio-temporal indicator results for the food web assessment and the Atlantis is useful for strategic analyses at a system level and for testing whether observed trends in ecosystem components can be reproduced (Fulton et al. 2011; Bossier et al. 2021). Piroddi et al. (2015) identified EwE models as most widely used food web models in Europe: in the North Sea, the Celtic Seas, the Bay of Biscay, the Baltic Sea, the Mediterranean Sea and the Black Sea. Because of this wide use, this section gives an example of the possibilities of EwE models to support the food web assessment.

EwE is an opensource, freely available modeling framework comprising three modules: (i) Ecopath—a static, mass-balanced snapshot of the system, (ii) Ecosim—a time dynamic simulation module and (iii) Ecospace—a spatial and temporal dynamic module (www.ecopath.org; Christensen and Walters 2004). The Ecopath input data are trophic interactions expressed as diet composition, estimates of biomass, production and consumption rates, and mortality. The Ecosim as a temporal model, requires input data as a time series of environmental and anthropogenic forcing. Ecospace employs the spatial time-dynamic model in each cell of the raster grid, while accounting for cell connectivity and species movements explicitly depended on environmental condition. The module allows for exploring effects and interplay of extrusive drivers on the ecosystem.

In a balanced model, the Ewe can predict biomass of any taxa that has the necessary population parameters but missing or incomplete time series data. The EwE software also provides ready-made ‘ecological indicators’ which relate to biomass of the model’s functional groups, diversity and ecosystem structure. Good state of the ecosystem or a functional group can be tested, for instance, by simulation scenarios or optimizing objectives for, e.g., ecosystem structure or ‘health’ (Christensen 1995). Pressures can be introduced to affect any of the population parameters which influence the model outcomes. The pressure impacts and setting of thresholds can be analyzed at level of functional groups by calculating ecological network analysis, indicating resilience, i.e. the system’s reserves before collapse (Heymans et al. 2014).

## Requirements from models to support food web assessments

Trophic guilds form the food web through dynamic interactions and, hence, GES of food webs cannot be assessed by one trophic guild alone but by an indicator comparing more than one guild at the same time. This entails several problems for the current definition of indicator thresholds. First of all, it is likely that the current indicators’ GES thresholds have not been aligned with each other, as they were developed in isolation. Moreover, there is probably more than one food web configuration that maintains GES at different biomass-levels of one single guild (cf. Yletyinen et al. 2016). The GES harmonisation and the alternative stable states could be evaluated using models, leading possibly to better-informed GES thresholds than what can be inferred from monitoring data alone. Simulation models that can be run with different starting points can be used to find low and high limit values for species and guild abundances that are still consistent with healthy and stable ecosystem functioning. Because GES assessments under the MSFD require an indicator-based approach (EU 2017), we therefore argue that support from food web models can substantially improve this approach.

While potential benefits of food web models are clear, their realization in practise depends strongly on model limitations and data needs. Uncertainties can stem from the model structures, e.g. the predator–prey relationships (e.g. Tunney et al. 2017), as missing trophic connections potentially lead to erroneous outputs when simulating yet-unseen scenarios. Additionally, the model parameters, e.g. the feeding, growth, or recruitment rates, are usually assumed to follow functions that are invariant in time. This assumption is usually needed to derive these parameters from data, but it may be problematic in the face of regime shifts and changing climates and other stressors (but see Tomczak et al. 2013). The Dynamic Bayesian networks models reviewed in this study (Uusitalo et al. 2018; Maldonado et al. 2019) explicitly allow the possibility that the trophic interaction functions change (implemented through latent variables in the models). This allows not only for guild balance to shift from one stable state to another along the defined functions, but also for species interaction functions (such as productivity for D4C4) to actually change, as could happen due to climate change, new unmodelled invasive species, or some other driver outside the modelled domain. These models also include an assessment of uncertainty of the model parameters and predictions. We did not further explore the Dynamic Bayesian networks models in this study, because more commonly used food web models may be the first step in supporting marine food web assessments.

## Recommendations for the next generation marine food web assessments

Current assessments of Baltic Sea food webs utilize indicators which are only partly fit for purpose. Among the 60 indicators, we found only nine clearly assessing the balance between trophic levels or guilds and only one potentially meeting the requirement of using at least three trophic guilds, with two non-fish trophic guilds and at least one primary producer trophic guild (sensu EU 2017). However, we believe that the available suite of food web indicators for the Baltic Sea has the potential to better meet the MSFD requirements if separate indicators are first rearranged from a taxonomic grouping into trophic guilds, then combined to indicate balance between the guilds (in the case of D4C2) and GES thresholds are re-defined for these combined indicators. We also found that there are major gaps for key trophic groups—especially macrophytes, macrozoobenthos and benthic planktivores (e.g. mysids)—which are driving forces for food web interactions in both the shallow coastal areas and open sea systems (Kiljunen et al. 2020; Kortsch et al. 2021). Our conclusion is that even after the proposed re-adjustments, there will be gaps in key indicators and needs for improving definitions of GES.

Food web models have great potential to support food web assessments where indicators fail (Piroddi et al. 2015), but they also have their model specific limitations. Our analysis of the available food web related models indicated that there are food web models that encompass multiple trophic levels and allow runs of different scenarios (e.g. management of pressures, re-adjustment of GES thresholds). Especially the EwE, Atlantis and Dynamic Bayesian networks models could provide valuable support to indicator-based assessments and EBM of human activities.

We recommend that models could support food web assessments in two overarching ways: First, the models can help to define GES thresholds for indicators by exploring their possible stable states under different pressure scenarios. We believe that such ‘intercalibration’ is difficult for separate indicators without a model framework (see however, Kauhala et al. 2019). Based on our analysis, we think that the simulations by EwE and Atlantis models could be currently the best choices to find GES thresholds for indicators because they transparently account for pressures-species interactions in the ecosystem and are readily available for many marine regions. Second, we highlight that the models help to populate food web indicators with data, if monitoring data is poor in time and/or space. This would be a more ecologically justified way to assess the diversity, biomass or size/age distribution of a trophic guild or taxon rather than simple interpolation of data, because the modelling approach considers the ecological interactions in the system. Integration of indicators and modelling outputs may give a strong assessment result, if implemented by integrated assessment frameworks (Borja et al. 2016; HELCOM 2018).

These two proposed approaches would capitalize on the strengths of ecosystem models, i.e. the fact that they integrate a vast body of scientific knowledge about food web interactions and ecosystem functioning with quality-assured data, and that they explicitly account for interactions of multiple ecosystem components through the time series. However, practical implementation of a hybrid food web assessment with indicators and models is not immediately within our reach in the Baltic Sea, because the parameterized models have not been set in synchrony with indicators in space and time (i.e., assessment areas and periods). In the Baltic Sea, where the regional sea convention (HELCOM) is preparing for the next assessment of the state of the marine environment, we propose to select pilot assessment areas in order to test our approach and overcome the technical challenges of the integration. Despite the support from models, food web assessments should still rely on monitored data and transparent metrics as is the spirit of the policy assessments like the EU MSFD.

## Supplementary Information

Below is the link to the electronic supplementary material.

Supplementary file1 (PDF 303 kb)

Supplementary file2 (XLSX 49 kb)
