# Supplementary material for: Food web assessments in the Baltic Sea: Models bridging the gap between indicators and policy needs
Source: Ambio. 2022 Jan 29;51(7):1687–97. doi: 10.1007/s13280-021-01692-x (PMC9110573; doi:10.1007/s13280-021-01692-x)
Supplement: Supplementary file 1 — Supplementary file1 (PDF 303 kb) [file 13280_2021_1692_MOESM1_ESM.pdf]

Food web assessments in the Baltic Sea: models bridging the gap between indicators and policy needs

Samuli Korpinen, Laura Uusitalo, Marie C. Nordström, Jan Dierking, Maciej T. Tomczak, Jannica Haldin, Silvia Opitz, Erik Bonsdorff, Stefan Neuenfeldt

Corresponding author: Samuli Korpinen, Finnish Environment Institute, Latokartanonkaari 11,

00790 Helsinki, Finland; email: samuli.korpinen@syke.fi

Ambio, Electronic Supplementary Material

This supplementary material has not been peer reviewed.

Appendix A: Full lists of food web indicators and models and their evaluation results:

Table S1. How the taxa / groups found in the models were mapped to the trophic guilds.

Table S2. Full list of food web indicators reported by EU member states under the EU Marine Strategy Framework Directive (MSFD) in 2018 for Descriptor 4 (food webs), or developed within the Baltic Sea Marine Environment Protection Commission (HELCOM), or published in scientific literature.

Table S3. Evaluation of the food web indicators using the criteria by Tam et al. (2017, simplified).

Table S4. Food web models in the Baltic Sea categorized to trophic guilds (see Table S1).
